# Global research trends in MRI of temporomandibular disorders: a bibliometric study and visualization analysis via CiteSpace

**DOI:** 10.22514/jofph.2025.072

**Published:** 2025-12-12

**Authors:** Yu Luo, Mengqi Liu, Yujiao Jiang, Kangkang Ma, Zhiye Chen

**Affiliations:** ^1^Department of Radiology, Hainan Hospital of PLA General Hospital, 572013 Sanya, Hainan, China; ^2^School of Medical Imaging, North Sichuan Medical College, 637100 Nanchong, Sichuan, China; ^3^Department of Radiology, First Medical Center of PLA General Hospital, 100853 Beijing, China; ^4^School of Medical Imaging, Bengbu Medical University, 233030 Bengbu, Anhui, China

**Keywords:** Bibliometrics, Temporomandibular disorders, Magnetic resonance imaging, Artificial intelligence, Research trends

## Abstract

**Background**: To conduct a bibliometric analysis mapping the intellectual landscape and emerging trends in Magnetic resonance imaging (MRI) research of temporomandibular disorders (TMD), identifying knowledge gaps and future directions. **Methods**: A total of 1017 articles were retrieved from the Web of Science Core Collection (WOSCC) database from 1995 to 2024 using the search formula: TS (Topic Search) = (“Temporomandibular Disorders” OR “Temporomandibular Joint Disease” OR TMD) AND TS = (“Magnetic Resonance Imaging” OR MRI). Author/country collaboration network, co-citation analysis, keyword clustering, and burst detection were conducted via CiteSpace 6.4.R1 (Parameters: Time slice: 1 year; g-index *k* = 25; Log-Likelihood Ratio clustering). **Results**: Annual publications exhibited triphasic growth, peaking at 75 articles in 2022. The United States (160 articles, centrality = 0.22) dominated global collaborations, while Shanghai Jiao Tong University emerged as the most productive institution (30 articles). Key clusters revealed 15 clusters (*Q* = 0.4235, *S* = 0.7521), with core clusters including “juvenile idiopathic arthritis” and “deep learning”. Burst strength identified four major research frontier directions: analysis of pathological characteristics of diseases (morphology with a burst strength of 4.49; anterior disc displacement with a burst strength of 3.69); innovation in imaging examination methods (ultrasonography with a burst strength of 3.61; cone-beam computed tomography with a burst strength of 3.51); development of intelligent diagnostic technologies (diagnostic criteria with a burst strength of 12.09; deep learning with a burst strength of 5.81); and support for clinical management applications (management with a burst strength of 4.62). **Conclusions**: This study delineates an evolving research paradigm integrating Artificial Intelligence (AI)-driven diagnostics with multimodal imaging.

## 1. Introduction

Temporomandibular disorders (TMD) is a generic term for pain and dysfunction of the temporomandibular joint (TMJ) and the muscles of mastication [1]. TMD is a significant health burden worldwide, affecting around 5–12% of the population [2], with an overall prevalence of around 31% in adults/elderly people and a slightly higher incidence rate in women than men [3]. It is considered the most common cause of chronic non-dental orofacial pain [4, 5]. The most common TMD subtypes include pain-related disorders (such as myofascial pain and arthralgias) and TMJ disorders (mainly internal disorders and degenerative joint disease) [6].

Magnetic resonance imaging (MRI) is a non-invasive medical imaging technique that uses strong magnetic fields, radiofrequency waves and computer processing to create detailed images of internal body structures. Unlike X-rays or computed tomography (CT), MRI visualizes soft tissue and functional changes without ionizing radiation [7]. The diagnosis of TMD usually involves three consecutive steps. First, the clinical history and examination findings are categorized into broad diagnostic groups. Then, specific physical disorders within these groups are identified (leading to an Axis I diagnosis). Finally, individualization is performed by assessing the degree of functional impairment (both physical and psychological) caused by the disorder (resulting in an Axis II diagnosis) [8]. As clinical examination alone is not sufficient to reliably assess TMJ abnormalities, MRI has established itself as the gold standard for a comprehensive assessment of TMJ status. This modality provides unparalleled visualization of bony and soft tissue abnormalities that cannot be detected with conventional imaging techniques [9]. However, the research focus and limitations in this area remain unclear.

CiteSpace, a Java-based application, analyzes and visualizes trends in scientific literature, hotspots and frontier areas of research within a particular discipline or field of knowledge through bibliometrics, co-occurrence analysis and cluster analysis. In CiteSpace, Betweenness Centrality, Modularity *Q*, Silhouette *S* and Burstness are important analytical indicators. They provide information on the structure and development trends of research fields from different perspectives and provide valuable information for academic research. Among them, betweenness centrality is an indicator that measures the degree of involvement of a node in the network in the paths connecting any two nodes. Nodes with high betweenness centrality are marked with a purple outline, representing key factors that connect different research directions, facilitate knowledge flow, and promote interdisciplinary research collaboration. Modularity *Q*-value reflects the tightness and independence of node clustering in the network. When *Q* > 0.3, the connections between nodes within a module are close, while those between modules are weak. This helps identify different research topics or groups and understand the internal structure and organizational patterns of the research field. The silhouette *S*-value is an indicator used to evaluate clustering quality. The closer the *S*-value is to 1, the more reliable the clustering results are, and the more reasonable the division of different topics in the research field becomes. Burst strength reflects the sudden emergence and changing trends of research hotspots, which is helpful for identifying hot topics in the research field [10, 11]. In different mapping diagrams, nodes represent authors, institutions, countries, or keywords. The size of a node indicates its frequency of occurrence or citation count, while the color denotes the year of occurrence or citation. This study applies CiteSpace to map research hotspots and frontiers in MRI studies of TMD, advancing understanding of TMD imaging characteristics.

## 2. Methods

### 2.1 Search strategy

This study only conducts a bibliometric analysis of the literature and therefore does not require ethical review or Institutional Review Board (IRB) approval. To enhance representativeness and the reliability of the data, we selected literatures through the Science Citation Index Expanded (SCIE) of the Web of Science Core Collection (WOSCC). All relevant studies used the following keywords: TS (Topic Search) = (“Temporomandibular Disorders*” OR “Temporomandibular Joint Disease*” OR TMD) AND TS (Topic Search) = (“Magnetic Resonance Imaging*” OR MRI). And the detailed search strategy and selection process are shown in Fig. 1. Through an advanced search, we retrieved documents spanning the period from 01 January 1995 to 31 December 2024. A total of 1254 documents were searched out, and 1017 documents were retained after screening. All the literature in question was exported in a text-only file format and marked “full record and cited references”.

**Fig. 1.** Flowchart for the search and selection process of this bibliometric analysis.

### 2.2 Eligibility and ineligibility criteria

We included peer-reviewed, published original articles and reviews centered on MRI of TMD. Items excluded were: (1) conference abstracts or corrigenda; (2) unpublished manuscripts; (3) duplicate publications; (4) articles unrelated to the topic.

### 2.3 Bibliometrics and visualization analysis

We exported the retrieved papers in plain text format, encompassing full records and references, and labeled them “download_XXX.txt”. These files were subsequently imported into CiteSpace for additional analysis. In this study, CiteSpace version 6.4.R1 was employed for literature analysis, with the following core parameters configured: a time-slicing interval of 1 year was adopted to segment the dataset chronologically; nodes were selected using the g-index (with a threshold of *k* = 25), which gives more weight to highly cited articles and is used to select influential nodes in the network, ensuring the inclusion of high-impact entities; the Pathfinder algorithm combined with temporal slicing was applied for pruning to optimize network structure; cluster analysis was performed using the Log-Likelihood Ratio (LLR) method, which identifies statistically distinctive terms from the cited literature to generate cluster labels, offering more meaningful topic representation than frequency-based methods; and a clustering threshold of *c* = 0.15 was set to balance the granularity and interpretability of the clustering results.

Meanwhile, the scientometric analysis in this study adheres to the Preferred Reporting Items for Systematic Reviews and Meta-Analyses Extension for Scoping Reviews (PRISMA-ScR) framework to ensure methodological rigor.

## 3. Results

### 3.1 Annual publication volume analysis

A total of 1017 publications were ultimately included in the analysis, comprising 931 articles and 86 reviews. The line graph (Fig. 2) uses the publication year as the x-axis and the number of articles as the y-axis, visually presenting the changes in the development level of a particular field. Since 1995, we have found that there have been publications on TMD MRI research every year. With the exception of 1995, when the number of publications was less than 10, the number of publications in all other years exceeded 10, and it reached a peak of 75 in 2022. Over the past decade, publication volume has stayed at a high level, averaging 54 per year, indicating that researchers’ interest in MRI research on TMD has been increasing significantly in recent years.

**Fig. 2.** Trend chart of the annual publication volume (1995–2024).

### 3.2 Author collaboration network analysis

The merged co-authorship network comprises 835 nodes and 1026 links, and we selected all authors who have collaborations (Fig. 3). The co-authorship network highlights prolific authors and their collaborative relationships. In the three clusters shown in Fig. 3, Jaremko JJ (8 articles) collaborated with 9 authors, Cron RQ (8 articles) collaborated with 10 authors and Larheim TA (5 articles) collaborated with 11 authors. Although the number of their publications is not very high, they are authors who value collaboration and communication with other scientists, which is beneficial for the progress and development of this field of research. We also found that collaboration between authors in the three clusters is low, which could be due to the different research topics in the different clusters. In contrast, the most prolific authors are Emshoff R (24 articles in total), Rudisch A (21 articles) and Bertram S (19 articles), who only collaborated with 7 authors each, suggesting that their communication in this field is not very active.

**Fig. 3.** Visualized maps of bibliometric analysis involving author co-occurrence in the field of MRI related to TMD from 1995 to 2024. Node size represents publication volume; edge thickness indicates collaboration frequency; edges denote collaborative relationships. MRI: Magnetic resonance imaging; TMD: Temporomandibular disorders.

### 3.3 The country and institution cooperation network analysis

Selecting the top 15 institutions by number of publications, the merged institutional collaboration network is shown in Fig. 4A, which includes 441 nodes and 723 connections. The top five institutions are Shanghai Jiao Tong University (30 articles), Egyptian Knowledge Bank (EKB) (27 articles), Sichuan University (24 articles), University of Innsbruck (24 articles), and Seoul National University (SNU) (21 articles).

For the top 20 countries ranked by number of publications, the merged national collaboration network is displayed in Fig. 4B, containing 56 nodes and 237 connections. The United States leads with 160 articles, followed by Japan (148 articles), the People’s Republic of China (130 articles), Brazil (78 articles), and Germany (78 articles). However, countries such as New Zealand, Albania, Tunisia, Nigeria, and Hungary have only 1 publication each. The purple rings surrounding the circles in the graph signify a betweenness centrality exceeding 0.1. Betweenness centrality gauges the probability that any shortest route in the network traverses a specific node, functioning to evaluate the significance of each node within the graph. When the betweenness centrality value exceeds 0.1, the node is considered a key node. Among the top five countries by publication volume, all except Japan have a betweenness centrality exceeding 0.1, highlighting their significant contributions to this field.

**Fig. 4.** Visualized maps of bibliometric analysis involving institutions and countries in the field of MRI related to TMD from 1995 to 2024. Node size represents publication volume; edge thickness indicates collaboration frequency; purple rings denote high betweenness centrality (pivotal bridging roles). (A) The Network of Co-Institution. (B) The Network of Co-country. MRI: Magnetic resonance imaging; TMD: Temporomandibular disorders.

### 3.4 Author and journal co-citation network analysis

Fig. 5A shows the network of authors with a co-citation frequency exceeding 50. The top 10 most cited authors are Larheim TA (cited 303 times), Westesson PL (cited 289 times), Katzberg RW (cited 268 times), Tasaki MM (cited 258 times), Emshoff R (cited 217 times), Schiffman E (cited 200 times), Dworkin S (cited 185 times), Manfredini D (cited 153 times), Okeson JP (cited 127 times), and Ahmad M (cited 118 times). Although the top 10 authors have been cited more than 100 times, only Dworkin S has a betweenness centrality reaching 0.1, indicating his prominent role in this field (Fig. 5A).

And Fig. 5B shows the network of journals with a co-citation frequency exceeding 100. The top 5 most cited journals are Oral Surg Oral Med O, cited 737 times, followed by J Oral Maxil Surg (cited 679 times), Int J Oral Max Surg (cited 522 times), Radiology (cited 478 times), and J Oral Rehabil (cited 448 times) (Fig. 5B).

**Fig. 5.** Visualized maps of bibliometric analysis involving cited authors and cited journals in the field of MRI related to TMD from 1995 to 2024. Node size indicates co-citation frequency; edges denote co-citation relationships; edge thickness reflects co-citation strength. (A) The Network of Author Co-citation. (B) The Network of Journal Co-citation. MRI: Magnetic resonance imaging; TMD: Temporomandibular disorders.

### 3.5 References co-citation analysis

Fig. 6A and Table 1 display the leading co-cited references that are marked by high co-citation frequency and high betweenness centrality. The first co-cited reference, published by Schiffman E *et al*. [12], provides a new evidence-based diagnostic and treatment protocol for Dental Occlusion/Temporomandibular Disorders (DC/TMD), which is more suitable for clinical and research settings. Valesan LF evaluated the prevalence of TMD in the general population [2]. Tasaki MM investigated the prevalence of various types of TMJ disc displacement in patients and asymptomatic volunteers to develop a classification system for TMJ disc displacement [13]. The reference by Katzberg RW used MRI to determine the prevalence and specific anatomical types of disc displacement in asymptomatic and symptomatic subjects, potentially explaining the anatomical variations in abnormal disc positions [14]. Ahmad M established comprehensive imaging analysis criteria for the RDC/TMD validation project [15]. Tasaki MM investigated the prevalence of various types of TMJ disc displacement in patients and asymptomatic volunteers to develop a classification system for TMJ disc displacement [13].

**Table 1.** The top 10 references by citation frequency.

Rank	First author	Frequency	Centrality	Year	Cited references	Journal	IF
1	Eric Schiffman	52	0.07	2014	Diagnostic Criteria for Temporomandibular Disorders (DC/TMD) for Clinical and Research Applications: recommendations of the International RDC/TMD Consortium Network* and Orofacial Pain Special Interest Group^†^	J Oral Facial Pain Headache	1.9
2	Lígia Figueiredo Valesan	32	0.00	2021	Prevalence of temporomandibular joint disorders: a systematic review and meta-analysis	Clinical Oral Investigations	3.1
3	Mark M. Tasaki	28	0.03	1996	Classification and prevalence of temporomandibular joint disk displacement in patients and symptom-free volunteers	Am J Orthod Dentofacial Orthop	3.0
4	Richard W. Katzberg	26	0.02	1996	Anatomic Disorders of the Temporomandibular Joint Disc in Asymptomatic Subjects	J Oral Maxillofac Surg	2.3
5	Mansur Ahmad	26	0.03	2009	Research diagnostic criteria for temporomandibular disorders (RDC/TMD): development of image analysis criteria and examiner reliability for image analysis	Oral Surg Oral Med Oral Pathol Oral Radiol Endod	2.5
6	Risa Matsubara	19	0.04	2018	Assessment of MRI findings and clinical symptoms in patients with temporomandibular joint disorders	Dentomaxillofac Radiol	4.1
7	Tore A Larheim	19	0.11	2005	Role of magnetic resonance imaging in the clinical diagnosis of the temporomandibular joint	Cells Tissues Organs	1.9
8	Thomas J Vogl	18	0.04	2016	The value of MRI in patients with temporomandibular joint dysfunction: Correlation of MRI and clinical findings	Eur J Radiol	3.3
9	Thekla Von Kalle	18	0.31	2013	Contrast-enhanced MRI of normal temporomandibular joints in children—is there enhancement or not?	Rheumatology (Oxford)	4.4
10	Daniel Talmaceanu	17	0.03	2018	Imaging modalities for temporomandibular joint disorders: an update	Clujul Med	0

*MRI: Magnetic resonance imaging; IF: Impact Factor.*

Other highly cited references include the following: Larheim TA revealed the role of MRI in the clinical diagnosis of temporomandibular joints, concluding that MRI is the best method for diagnostic evaluation of TMJ conditions [9]. Matsubaraetal R concluded that disc displacement without reduction and high-grade joint effusion (an indicator of joint inflammation) are associated with TMD symptoms, suggesting that treatment strategies for disc displacement without reduction and reducing inflammation may alleviate clinical TMD symptoms [16]. Vogletal TJ found that MRI has highly acceptable specificity and sensitivity for diagnosing anterior disc displacement and osseous changes in the TMJ [17]. Additionally, MRI should primarily be used for severe, refractory cases and surgical planning purposes.

Fig. 6B displays the largest 19 clusters of co-cited references. Fig. 7 presents the top 20 references featuring the strongest citation bursts, signaling emerging trends or increasing focus in this field. Typically, the most frequently co-cited references are also prone to show the strongest citation bursts.

**Fig. 6.** Visualized maps of bibliometric analysis involving the top 20 cited references in the field of MRI related to TMD from 1995 to 2024. Node size indicates co-citation strength; edges denote co-citation relationships; edge thickness reflects co-citation frequency; purple rings highlight pivotal bridging references. (A) The Network of Co-cited References. (B) The Network of Co-cited References Clusters. MRI: Magnetic resonance imaging; TMD: Temporomandibular disorders.

**Fig. 7.** Top 20 References with strongest citation bursts. Burst strength indicates citation surge intensity; blue segments denote stable citation periods; red segments mark burst durations.

### 3.6 Keywords analysis

#### 3.6.1 Keyword co-occurrence analysis

Nodes stand for keywords, with the size of each circle reflecting the citation frequency of these keywords, and each keyword has a citation frequency of over 50 (Fig. 8A). The top ten most influential keywords based on citation frequency were identified (Table 2). High-impact keywords reflect hot topics in the field, while nodes marked with purple circles denote those with outstanding betweenness centrality. The top five keywords by citation frequency were temporomandibular joint (399 times), disorders (363 times), magnetic resonance imaging (304 times), internal derangement (233 times), and pain (178 times). In contrast, the keywords with the highest betweenness centrality were disease (0.13), MRI (0.11), abnormality (0.11), diagnosis (0.10), and association (0.10). This divergence highlights that while high-frequency keywords represent dominant research topics, those with elevated centrality serve as critical hubs bridging interdisciplinary concepts within the knowledge network.

**Table 2.** The top 10 representative keywords.

Rank	Keywords	Count	Centrality
1	Temporomandibular joint	399	0.03
2	Disorders	363	0.03
3	Magnetic resonance imaging	304	0.08
4	Internal derangement	233	0.06
5	Pain	178	0.06
6	Prevalence	171	0.08
7	Displacement	110	0.08
8	Temporomandibular disorders	108	0.06
9	Disc displacement	99	0.04
10	Temporomandibular joint	96	0.06

#### 3.6.2 Keyword cluster map analysis

To better visualize research frontiers in specific topics, keyword clustering analysis was performed. Spectral clustering algorithms were applied to group keywords, and the LLR method was used to extract labels for each cluster from cited literature. A modularity (*Q*-value) >0.3 suggests significant community structure, while a silhouette (*S*-value) >0.7 indicates robust and convincing clustering. A total of 15 clusters were ultimately retained: bone marrow edema, diagnostic value, temporomandibular joint arthralgia, juvenile idiopathic, comparative study, anterior disk displacement, internal derangement, biomechanical effect, voxel-based morphometry study, deep learning, hyaluronic acid, masseter muscle, symptomatic patient, correlation. The modularity *Q*-value of 0.4235 and silhouette *S*-value of 0.7521 (Fig. 8B) confirmed the reliability and academic significance of the clustering results. The overlapping color blocks in the cluster visualization indicate close interconnections and dynamic interactions among distinct thematic domains.

**Fig. 8.** Visualized maps of bibliometric analysis of keywords with an occurrence frequency of over 50 in the field of MRI related to TMD from 1995 to 2024. Node size indicates keyword co-occurrence frequency; edges denote co-occurrence relationships; edge thickness reflects co-occurrence strength; purple rings highlight pivotal bridging topics. (A) The Network of Co-occurring Keywords. (B) The Network of Co-occurring Keywords Clusters. MRI: Magnetic resonance imaging; TMD: Temporomandibular disorders.

#### 3.6.3 Keywords with citation bursts

Fig. 9 presents the top 59 keywords featuring citation bursts. The blue line stands for the time range, while the red line signifies the period when a keyword showed a citation burst [18]. The keyword with the strongest citation burst intensity was diagnostic criteria, followed by classification. Notably, after excluding the search subject terms, the citation bursts of association, involvement, ultrasonography, management, morphology, diagnostic criteria, anterior disc displacement, deep learning, network, and cone-beam computed tomography have persisted until 2024, highlighting their sustained academic relevance and emerging dominant position in current research trends.

**Fig. 9.** Top 58 keywords with strongest citation. Burst strength indicates citation surge intensity; blue segments denote stable citation periods; red segments mark burst durations.

## 4. Discussion

### 4.1 Research status

In this study, bibliometric methods were applied to analyze 1017 articles on MRI research of TMD from WOSCC. Quantitative analyzes were performed on journal metadata, including annual publication volume, authors, countries and institutions. In the period from January 1995 to December 2024, the number of published articles showed a general upward trend (Fig. 2), and this trend can be divided into three phases.

Phase 1 (1995–1998) marked the initial phase of research with limited publications and slow growth. Fundamental studies during this period created a solid theoretical basis for later advances. Phase 2 (1999–2007) saw moderate growth in publications, indicating a lack of groundbreaking advances. Phase 3 (2008–2024) represents the peak, characterized by a surge in publications, particularly with the fastest growth between 2015 and 2016, indicating accelerated development in the field. Over the past three decades, advances in novel MRI technologies have demonstrated significant scientific potential and clinical utility in this field. Nowadays, 3.0T MRI is widely used to improve the resolution of temporomandibular joint images [19]. Traditional sequences such as T1-weighted imaging (T1WI), T2-weighted imaging (T2WI), and proton density-weighted imaging (PDWI) still hold value in assessing disc displacement, soft tissue conditions, and joint effusion [20]. Fluid-attenuated inversion recovery (FLAIR) sequences are used specifically to differentiate between inflammatory processes and simple fluid accumulation in the joint space [21], while fat saturation techniques improve the detection of joint adhesions and fibrotic changes [22]. Dual Echo Steady State (DESS) combines the signals from Fast Imaging with Steady-state Precession (FISP) and Phase Sensitive Inversion Recovery Fast Imaging with Steady-state Precession (PSIF) echoes, which enhances T2 specificity, reduces signal decay caused by dephasing, and achieves excellent visualization of the temporomandibular joint [23]. Quantitative analysis using apparent diffusion coefficient (ADC) values derived from diffusion-weighted imaging (DWI) provides an objective assessment of the severity of masseter muscle myalgia in TMD patients [24]. Diffusion Tensor Imaging (DTI), as a non-invasive method, has gained increasing attention. It can be used to characterize the morphological features and internal structural arrangement of masticatory muscles and can detect early musculoskeletal changes [25]. Chemical shift-encoded MRI (CSE-MRI) is a method capable of quantifying proton density fat fraction (FF), which enables quantitative analysis of the masticatory muscles in patients with TMD through fat fraction [20]. Ultrashort echo time (UTE) sequence improves the visualization of short T2 structures, including the fibrocartilaginous disc and the fibrocartilage of the inner wall of the articular surface of the joint [26].

Author visualization analysis (Fig. 3) revealed that the most prolific authors in TMD-MRI research were Emshoff R, Rudisch A and Bertram S, underscoring their prominent contributions. At the national level, the United States, Japan, and the People’s Republic of China produced the greatest number of publications (Fig. 4B). Notably, the U.S. dominated in both publication volume and betweenness centrality, highlighting its pivotal role in fostering global integration and knowledge exchange. In contrast, the observed dichotomy between Japan’s high publication output (116 articles) and low betweenness centrality (0.1) suggests a potential “scientific silo” phenomenon, wherein domestic research networks may prioritize local collaboration over international knowledge exchange. We also note that although South Korea ranks 9th in the number of publications (50 articles), Seoul National University in South Korea has published 21 articles with a betweenness centrality of 0. This result indicates that institutions in South Korea, including Seoul National University, not only need to further strengthen research in this area in the future, but should also emphasize establishing close cooperative relationships with other institutions worldwide to promote the global development of this research area. In contrast, there are only 11 publications in Denmark. Aarhus University in Denmark has published 9 articles, with its betweenness centrality of 0.15 being the highest among the institutions. This indicates that Aarhus University is not only a leading institution in this field domestically, but also very active in international exchange and collaboration. Collaboration with countries or institutions specializing in this field could enhance cross-border understanding and drive progress in TMD-MRI research. We advocate for deeper international and interdisciplinary cooperation to dismantle academic barriers and advance this domain. Ten countries represented by New Zealand have only 1 publication each, suggesting that these countries not only need to increase their focus in this area, but also improve international exchange and cooperation.

We analyzed the prominent representatives among the cited references by referring to citation counts, betweenness centrality, and citation bursts. The article on TMD diagnosis and treatment published by Schiffman E in 2014 had the highest citation count in the sample of this study. It exhibited the strongest burst intensity, reaching 25.79, during the three-year period from 2016 to 2019. This article primarily updated the Axis I diagnostic algorithm of the Research Diagnostic Criteria for Temporomandibular Disorders (RDC/TMD), established MRI as an essential auxiliary tool, provided methodological support for TMD-related MRI research, and facilitated the development of TMD imaging diagnostic criteria and clinical-imaging correlation models [12]. The reference with the highest betweenness centrality was published by Von KT in 2013. Its findings confirmed the presence of physiological enhancement in the TMJ of normal children, offering a crucial reference for distinguishing pathological enhancement in children with juvenile idiopathic arthritis. This contributed to optimizing MRI assessment protocols for juvenile idiopathic arthritis children and resolved the controversy over the degree of contrast in the TMJ of normal children [27]. Additionally, we noted that in Fig. 7, the cited reference that has maintained a continuous burst in recent years with a burst intensity exceeding 10 is the 2021 publication by Valesan LF. Its significance lies mainly in evaluating the two diagnostic criteria (RDC/TMD and DC/TMD), determining the prevalence of temporomandibular joint disorders in the general population, and addressing the controversy over the actual prevalence of TMD in the population, which arose due to the lack of homogeneity in diagnostic criteria used in previous surveys.

### 4.2 Research hotspots and frontiers

Clustering of citations (Fig. 6B) is generally regarded as the foundation of the current knowledge system, while clustering of keywords (Fig. 8B) is the focus of attention of current researchers. Therefore, we analyzed and compared these two clusters together, which helps us to better grasp the hotspots of research. This field has made a leap from qualitative to quantitative analysis. For example, Jeon *et al*. [28] were the first to perform quantitative analysis of fat content in the masseter muscles of TMD patients using chemical-encoded magnetic resonance imaging, which is expected to become a biomarker for objective assessment of muscles. For example, Li *et al*. [29] investigated changes in the whole brain gray matter volume and cerebral blood flow in TMD patients using MRI. Finally, juvenile idiopathic arthritis is also recognized as an important research focus. Juvenile idiopathic arthritis is usually associated with morphological changes of the TMJ, but patients with this type often have no clinical symptoms, and their prognosis is not favorable. Therefore, MRI is of great importance as the gold standard for the early diagnosis and treatment of this disease [30].

Keyword citation burst strength reflects emerging research hotspots; thus, we will conduct analysis from four aspects: analysis of pathological characteristics of diseases, innovation in imaging examination methods, development of intelligent diagnostic technologies, and support for clinical management applications.

Morphological changes in TMD have long been a focus of research. Anterior disc displacement is the most common subtype of TMD, leading to clinical symptoms such as malocclusion, mandibular retrognathia and facial asymmetry, and can cause severe psychosocial problems for patients [31]. MRI is considered the gold standard for assessing the morphology and location of articular disc [32]. However, the complexity of MR image analysis of the TMJ is increased by the often-unclear description and low contrast of the components of the TMJ and surrounding tissues, as well as changes in the morphology of the disk due to disease progression [31]. Increased research in this area will improve diagnostic accuracy and reproducibility in this area. Secondly, some studies have shown that pain in TMD patients may be related to central sensitization. Although central sensitization cannot be measured directly, it can be assessed in terms of amplitude, duration or spatial extent using functional magnetic resonance imaging [4], which provides technical support for biomarker research.

MRI, the gold standard for the diagnosis of TMD, has limited use for technical, economic and social reasons [33], paving the way for the development of other cost-effective and convenient imaging examinations. Both CBCT and MRI can accurately measure joint space and condylar morphologic changes, although both have different advantages—CBCT is superior in detecting bony abnormalities and is often the first choice for diagnosing bony lesions, while MRI is superior in detecting soft tissue [34]; a study shows that ultrasound achieves a specificity of 90% and a sensitivity of 80% in the diagnosis of disc displacement, and its advantages in real-time capability and cost-effectiveness make it promising [35]. Although ultrasound may have clinically acceptable diagnostic accuracy in diagnosing disc displacement of TMJ, due to existing disagreements regarding the standardization of relevant procedures, additional training for physicians in operation and interpretation is still required to ensure diagnostic accuracy [36]. Combining MRI with other imaging examinations into a multimodal examination technology will facilitate the improvement of diagnostic criteria and thus increase the accuracy and reliability of existing diagnoses.

Although the DC/TMD diagnostic criteria are the most widely used, they have limitations, such as limited diagnostic accuracy, sensitivity and specificity. Therefore, there is an urgent need for new diagnostic tools to improve the efficiency and precision of TMD diagnosis [37]; in recent years, artificial intelligence has gained popularity in various medical fields, with AI models such as U-Net, RetinaNet, MobileNetV2, ResNet, InceptionV3 and VGG16 ensemble playing an important role in segmentation and interpretation of anatomical structures across different imaging modalities, achieving accuracy of more than 83%, while MobileNet V2 and ResNet have better performance metrics, showing their reliability in the diagnosis of TMD [38]; at the same time, we noted that all studies use locally available samples from a single center, which may lead to overfitting and only apply to the population for which they were trained. To improve the validity and universality of the models, further research examining more diverse multicenter data will be the next research focus [39].

The etiology of TMD is multifactorial, with the exact causes of symptoms being difficult to identify and the intensity of TMD symptoms varying; therefore, management for TMD patients should be individualized. However, due to controversies in the literature about TMD diagnosis and management protocols, the choice of treatment methods is often strongly influenced by the expertise of the treating physicians [40]. The development of uniform diagnostic and management protocols will standardize treatment approaches for patients, thereby reducing the medical risks associated with inconsistent standards.

## 5. Limitations of this study

The limitations of this study are mainly as follows: (1) Database bias: this study only included English literature from WOSCC, which may carry risks of omitting distinctive research from non-English-speaking countries and studies in other databases. (2) Differences in economic strength and population size among countries may affect research development, potentially introducing bias [41]. (3) The visualization tools used have limitations, as they cannot exclude self-citations, which may lead to bias. (4) Since some excellent newly published articles may be omitted due to delays, the results of this study may suffer from insufficient timeliness.

## 6. Conclusions

This study systematically shows the research development and recent trends in the field of MRI in TMD from 1995 to 2024 through a bibliometric analysis of CiteSpace. Key findings show that the volume of research in this field has continued to increase, with the number of publications peaking at 75 articles in 2022 and an average of 54 articles per year over the last decade, confirming the irreplaceability of MRI as the gold standard for the assessment of TMD. The network of national collaborations shows that the United States (160 publications, betweenness centrality 0.22), China (130 publications) and Brazil (78 publications) form the core of the global collaboration. However, Japan (148 publications, betweenness centrality 0.05) and South Korea (50 publications, betweenness centrality 0) show the phenomenon of “academic isolation”, which is characterized by high output but low centrality and underlines the need to strengthen international collaboration. Citation burst detection further reveals four major frontier transitions: analysis of pathological characteristics, innovation in imaging examination methods, development of intelligent diagnostic technologies, and support for clinical management applications.

Future directions should prioritize: (1) developing AI models based on multi-center big data to enhance the validity and universality of the models; (2) promoting interdisciplinary innovation to explore the comorbidity mechanisms between TMD and systemic diseases, thereby advancing personalized treatment strategies; (3) combining MRI with other imaging examinations to form multimodal examination technology, so as to improve the accuracy and reliability of existing diagnoses. Addressing these priorities will require concerted efforts to bridge the current translational gap between technical innovation and clinical implementation.
